# Effects of MCC950 on lipid profiles, glycemic control, and sympathetic dysfunction in a streptozotocin-induced type 1 diabetic rat model

**DOI:** 10.3325/cmj.2026.67.285

**Published:** 2026-08

**Authors:** Shamala Devi Subramaniam, Xin Yi, Rajesh Ramasamy, Nurul Izzati Uda Zahli, Siti Saleha Masrudin, Razif Abas

**Affiliations:** 1 Department of Medical Microbiology, Faculty of Medicine and Health Sciences, Universiti Putra Malaysia, Serdang, Selangor, Malaysia; 2 Department 1 of Cardiovasology, North China University of Science and Technology Affiliated Hospital, Tangshan City, Hebei Province, China; 3 Department of Pathology, Faculty of Medicine and Health Sciences, Universiti Putra Malaysia, Serdang, Selangor, Malaysia; 5 Department of Veterinary Pathology & Microbiology, Faculty of Veterinary Medicine, Universiti Putra Malaysia, Serdang, Selangor, Malaysia; 5 Department of Human Anatomy, Faculty of Medicine and Health Sciences, Universiti Putra Malaysia, Serdang, Selangor, Malaysia

## Abstract

**Aim:**

To evaluate the effects of MCC950, a selective NLRP3 inflammasome inhibitor, on glycemic control, lipid profiles, and sympathetic dysfunction in a streptozotocin (STZ)-induced type 1 diabetes mellitus (T1DM) rat model.

**Methods:**

Male Wistar rats were rendered diabetic by intraperitoneal injection of STZ (50 mg/kg). T1DM induction was confirmed by persistent hyperglycemia, reduced serum C-peptide, and pancreatic histopathological changes. Diabetic cardiac autonomic neuropathy was established by elevated serum noradrenaline (NA) before treatment. Diabetic rats subsequently received MCC950, insulin, or normal saline for four weeks. Fasting blood glucose, serum lipid profiles, C-peptide, pancreatic histology, and serum NA were evaluated before and after treatment.

**Results:**

MCC950-treated rats showed significantly reduced fasting blood glucose, total cholesterol, triglycerides, and LDL-cholesterol (all *P* < 0.0001) levels compared with untreated diabetic rats, while HDL levels remained unchanged, which suggests an improvement in diabetes-associated dyslipidemia. MCC950 treatment also partially restored C-peptide concentrations, improved pancreatic histology, and reduced serum NA levels.

**Conclusion:**

These findings suggest that NLRP3 inflammasome inhibition may improve metabolic disturbances associated with experimental T1DM. Further studies incorporating direct functional assessments of cardiac autonomic function are required to determine its role in diabetic cardiac autonomic neuropathy.

Diabetic cardiac autonomic neuropathy (DCAN) is a serious and often overlooked complication of diabetes mellitus (DM), characterized by dysfunction of the autonomic nervous system innervating the heart (1). It is associated with an increased risk of arrhythmias, sudden cardiac death, and overall mortality (2). Despite advances in diabetes management, DCAN remains a considerable clinical challenge, with limited treatment options. Dyslipidemia, a common metabolic abnormality in diabetes, has been implicated in the pathogenesis of DCAN, further complicating the management of this condition (3). However, there is a lack of targeted interventions capable of simultaneously addressing dyslipidemia and DCAN in diabetes.

Noradrenaline (NA) is intricately involved in the development and progression of DCAN in rats (4). As a key neurotransmitter in the sympathetic nervous system, it plays a pivotal role in regulating cardiovascular function (5). In the context of diabetes, dysregulated NA signaling pathways lead to aberrant sympathetic activity, endothelial dysfunction, and oxidative stress, all of which contribute to the pathogenesis of DCAN (6). Rat models of diabetes have demonstrated altered NA levels and adrenergic receptor expression in the cardiac autonomic nervous system, highlighting the importance of NA in mediating the structural and functional changes observed in DCAN (7,8). Understanding the relationship between NA and DCAN in rats may provide valuable insights into potential therapeutic targets for mitigating the progression of diabetic cardiovascular complications.

The nucleotide-binding oligomerization domain-like receptor protein 3 (NLRP3) inflammasome has emerged as a potential therapeutic target in the treatment of various inflammatory and metabolic disorders, including diabetes and dyslipidemia (9). MCC950, a selective inhibitor of the NLRP3 inflammasome, has shown promise in preclinical studies for its ability to attenuate inflammation and improve metabolic parameters (10). However, its specific effects on DCAN and dyslipidemia, particularly in the context of type 1 diabetes mellitus (T1DM), remain largely unexplored. Therefore, the aim of this study was to evaluate the effects of MCC950 on glycemic control, lipid metabolism, pancreatic β-cell function, and serum NA as a biomarker of sympathetic dysfunction in a streptozotocin (STZ)-induced T1DM Wistar rat model.

## MATERIAL AND METHODS

### Experimental animals

**Figure 1:**
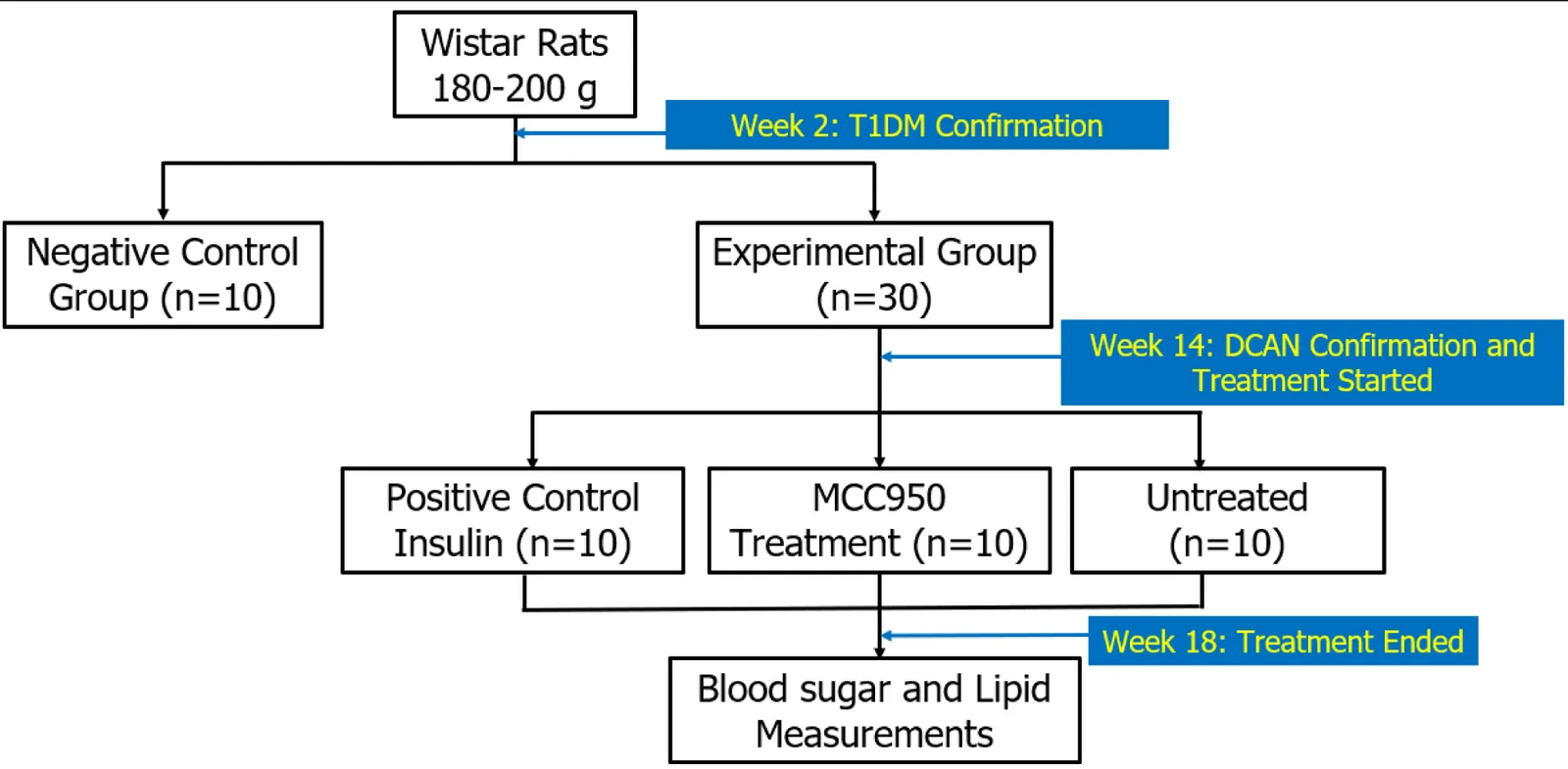
The study flowchart. Abbreviations: T1DM – type 1 diabetes mellitus; DCAN – diabetic cardiac autonomic neuropathy.

A total of 40 male Wistar rats, aged 6 weeks and weighing between 180 and 200 g, were sourced from the Bistari Enterprise (Taman Sri Serdang, Seri Kembangan, Selangor, Malaysia). Ethical clearance for animal handling was obtained from the Institutional Animal Care and Use Committee. The rats were housed in pairs in sanitized cages under regulated temperature and humidity levels, and a 12-hour light/dark cycle (11). They had unlimited access to standard rat pellets. The rats were allowed to adjust to the new environment for one week before the experiments commenced. The study flowchart is shown in Figure 1. All experimental protocols were approved by the Institutional Animal Care and Use Committee of Universiti Putra Malaysia. This study complies with the Animal Research: Reporting of In Vivo Experiments (ARRIVE) guidelines.

### T1DM establishment

After T1DM was confirmed, the rats were randomly divided into the negative control group (n = 10) and the T1DM group (n = 30). The T1DM group was subjected to intraperitoneal injections of 50 mg/kg STZ (12). Until the onset of DCAN, diabetic rats were kept under standard dietary and housing conditions without any treatment.

Week 0 was defined as the baseline, immediately before STZ injection. Blood glucose was measured at the following time points: weeks 0, 2, 14, 15, 16, 17, and 18. Before each blood glucose assessment, rats were fasted from 20:00 the preceding evening. Blood samples were collected from the tail after sterilization with an alcohol swab. A small segment (approximately 3 mm) of the tail tip was removed, and the blood glucose test paper was inserted into the Accu-Chek Guide Meter (Roche Diabetes Care, Inc., Indianapolis, IN, USA) for analysis (13).

### Confirmation of serum NA levels and DCAN establishment

NA in serum samples was measured using a commercially available competitive enzyme-linked immunosorbent assay kit (ELISA, Elabscience Biotechnology Co., Ltd, Wuhan, China), following the manufacturer's instructions (14). Blood samples were collected by tail venipuncture at two pre-treatment time points: week 10 and week 14, and immediately transferred into disposable microtubes for biochemical evaluation of sympathetic nervous system activity (12).

Whole blood was allowed to clot at room temperature. Serum was separated by centrifugation and stored at −80 °C until analysis. NA concentrations were measured using a competitive ELISA format, in which NA in the sample competes with a biotinylated NA derivative for binding to a limited number of NA-specific antibody sites (15). Fifty microliters of serum from each experimental group were added to the wells of a pre-coated 96-well ELISA plate, followed immediately by 50 μL of biotinylated detection antibody working solution. The plate was sealed and incubated for 45 minutes at 37 °C. After incubation, wells were aspirated and washed three times with wash buffer. Subsequently, 100 μL of horseradish peroxidase (HRP)-conjugate working solution was added and incubated at 37 °C for a further 45 minutes, followed by five wash cycles. Ninety microliters of TMB substrate solution was then added to each well and incubated for 15 minutes at 37 °C in the dark. The reaction was terminated with 50 μL of stop solution, which produced an immediate color change from blue to yellow. Optical density (OD) was measured at 450 nm within 15 minutes, and NA concentrations were determined against a standard curve generated according to kit-supplied standards (14).

### MCC950 treatment

Once the presence of DCAN in the experimental group was verified through the elevated NA levels (12), the experimental animals were randomly divided into three subgroups: positive control (n = 10), treatment group (n = 10), and untreated group (n = 10). The original control group maintained its role as the negative control. The positive control group received intraperitoneal (IP) insulin 5.0 U/kg/d (16), the treatment group received IP MCC950 3 mg/kg/d (17), and the untreated group received 5 mg/kg normal saline. The insulin-treated group served as a clinically relevant comparator for assessing the metabolic effects of MCC950 on the study outcomes rather than a specific therapeutic control for DCAN. All experimental groups received treatment for 28 days starting at week 14.

### Confirmation of serum C-peptide and pancreatic β-cell function

C-peptide was measured with an ELISA kit following the manufacturer's protocol. C-peptide is a reliable biomarker of endogenous insulin secretion and pancreatic β-cell function, as it is co-secreted in equimolar amounts with insulin but is not subject to hepatic first-pass metabolism (18). C-peptide measurement therefore provides an accurate assessment of residual β-cell function and is widely used to validate T1DM induction in animal models (12,19).

Blood samples were obtained by tail venipuncture at two time points: pre-treatment (weeks 0-14) and post-treatment (weeks 15-18), and placed into disposable microtubes for biochemical analysis (20). Serum was separated by centrifugation and stored at −80 °C until analysis. C-peptide concentrations were measured using a competitive ELISA format. Fifty microliters of serum from each experimental group were added to the wells of a pre-coated 96-well ELISA plate, immediately followed by the addition of 50 μL of biotinylated detection antibody working solution. The plate was incubated for 30 minutes at 37 °C to allow antigen-antibody binding. After incubation, wells were aspirated and washed three times with wash buffer. Subsequently, 100 μL of HRP-conjugate working solution was dispensed into each well, and the plate was incubated at 37 °C for 30 minutes, followed by five wash cycles. Next, 50 μL of Chromogen Solution A and 50 μL of Chromogen Solution B were added to each well, mixed gently, and incubated for 15 minutes at 37 °C in the dark for color development. The reaction was stopped by adding 50 μL of stop solution, which produced an immediate color change to yellow. OD was measured at 450 nm using a microplate reader, and C-peptide concentrations were determined by correlating sample OD values against the standard curve.

### Histological evaluation of pancreatic islet morphology and exocrine tissue integrity

Pancreatic tissue sections were examined for pathological changes in both the exocrine and endocrine compartments. Particular attention was given to the morphological integrity of acinar cells, which constitute the exocrine pancreas and are responsible for digestive enzyme secretion, and to the islets of Langerhans, the endocrine functional units responsible for insulin and glucagon production. Histopathological features assessed included acinar cell vacuolation and atrophy, the degree of interstitial and periductal fibrosis, ductal dilation, inflammatory cell infiltration, and the structural preservation and cellular composition of the islets of Langerhans. A minimum of five fields per section was evaluated across the mid-portions of the pancreatic tissue. Histopathological changes were graded according to the semiquantitative scoring criteria (Table 1).

**Table 1:** Semiquantitative scoring criteria for evaluating histopathological changes in pancreatic tissue sections

Grade	Histological criteria
0	Normal islet morphology, no infiltration
1	Mild disruption, occasional inflammatory cells
2	Moderate disruption, partial islet loss
3	Severe disruption, extensive infiltration

### Lipid profile assay

At week 14, after DCAN confirmation, and at week 18, after the 4-week treatment, whole-blood samples were collected using disposable test tubes, according to the manufacturer’s instructions (Premier Integrated Labs, Kuala Lumpur, Malaysia). Blood was collected from the tail in plain tubes, allowed to coagulate at room temperature, and transported in an ice storage container. Serum was used to assay total cholesterol (TC), high-density lipoprotein (HDL), low-density lipoprotein (LDL), and triglycerides (TG).

### Statistical analysis

Continuous data are presented as means ± standard deviations. The normality of distribution was assessed using the Shapiro-Wilk test. Differences between groups were assessed with an independent-samples *t* test or a Mann-Whitney U test. One-way analysis of variance (ANOVA) with a Tukey’s *post hoc* test was used for multiple group comparisons. A *P* value of less than 0.05 was considered significant. Statistical analysis was performed with GraphPad Prism software, version 10.0 (GraphPad Software, San Diego, CA, USA).

## RESULTS

### The effects of MCC950 on fasting blood glucose

Hyperglycemia was confirmed by week 2, after 1 week of acclimatization. As expected, glucose levels were normal in the negative control group (n = 6), and elevated in the experimental group (n = 30). Glucose levels remained significantly elevated until week 18.

**Figure 2:**
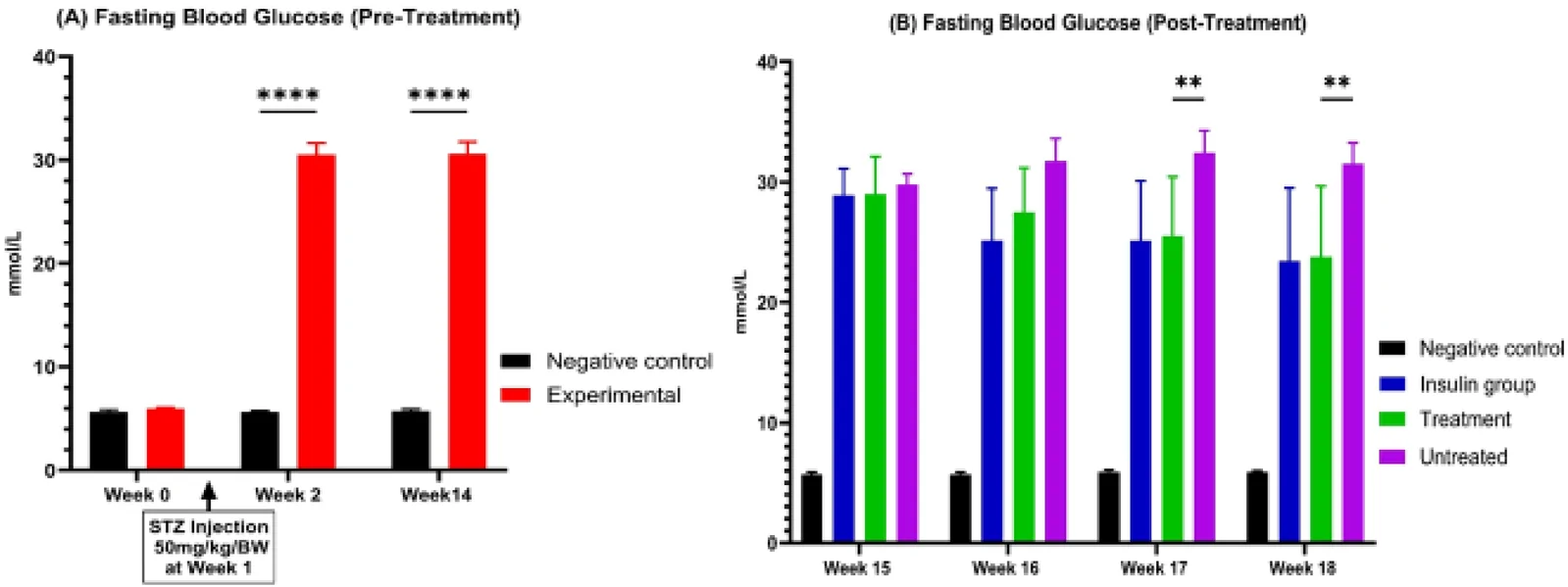
Fasting blood glucose levels before (**A**) and after treatment (**B**). Streptozotocin (STZ) was administered in week 1. Blood glucose levels significantly increased from week 2 onwards and remained elevated until week 16. At post-treatment at week 18, both the positive control (insulin) and treatment (MCC950) groups showed significantly reduced blood glucose level compared with the untreated group; **P < 0.001 and ****P < 0.0001.

All three experimental groups consistently showed higher glucose levels than the negative control group, even after treatment initiation. By week 18, both the insulin and MCC950 groups had significantly lower glucose levels than the untreated group. However, glucose levels did not significantly differ between the insulin and MCC950 groups (Figure 2).

### The effects of T1DM and MCC950 on serum NA

**Figure 3:**
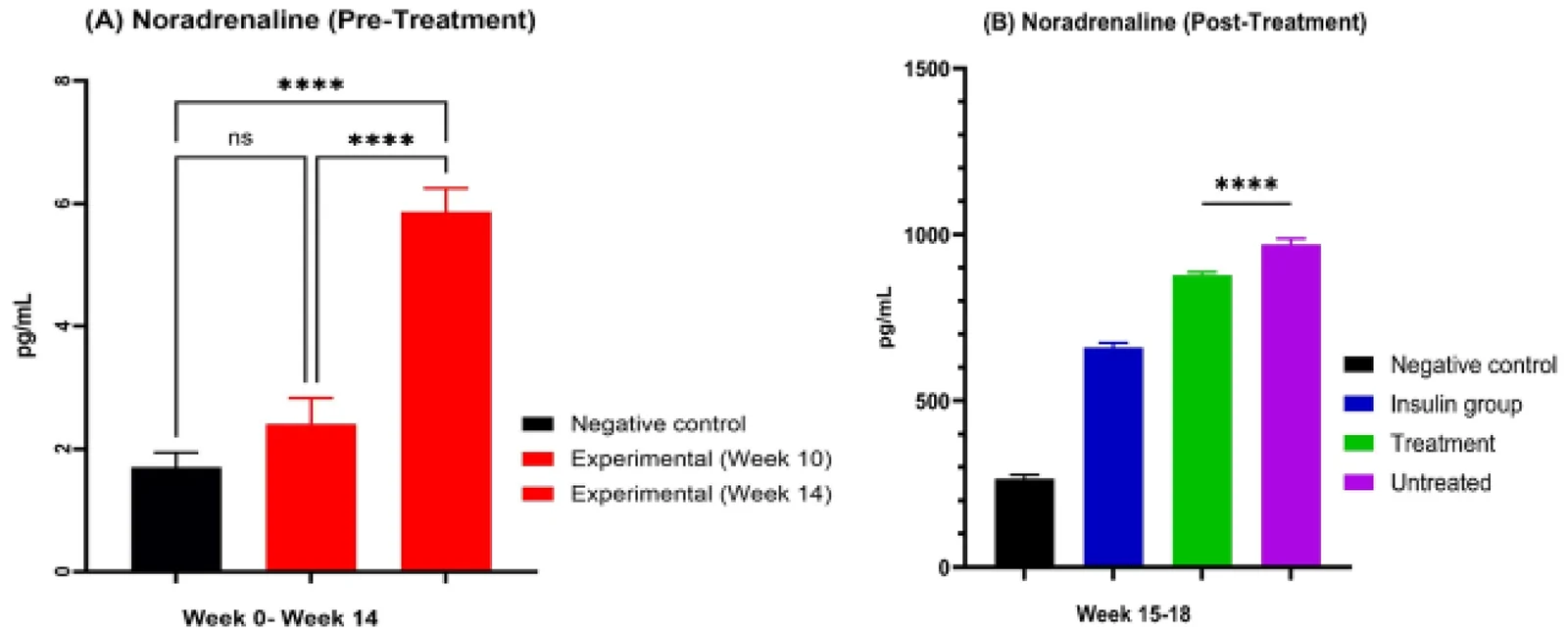
Noradrenaline levels before (**A**) and after treatment (**B**). Serum noradrenaline levels significantly increased by week 14, which indicated the establishment of diabetic cardiac autonomic neuropathy. No additional measurements were conducted at weeks 16, 17, and 18 due to the limited availability of chemicals (ns = no significant, ****P < 0.0001).

Serum NA concentrations were quantified at week 10 and week 14 to monitor progressive sympathetic dysregulation before treatment initiation (Figure 3, left panel). At week 10, the experimental group demonstrated a modest but non-significant elevation in serum NA compared with the negative control (2.4 ng/mL vs 1.77 ng/mL; ns), which suggested early but not yet fully established sympathetic overactivation.

By week 14, serum NA in the experimental group increased to 4.9 ng/mL, which was significantly higher compared with both the negative control (*P* < 0.0001) and the week 10 experimental values (*P* < 0.0001). The negative control group maintained stable baseline NA concentrations across both time points, reflecting normal autonomic regulation. These findings confirmed progressive sympathetic nervous system overactivation between weeks 10 and 14 and validated the establishment of DCAN before the beginning of treatment.

At weeks 15-18, NA was significantly different between all groups (*P* < 0.0001; one-way ANOVA with Tukey's *post-hoc* test) (Figure 3). NA concentration was highest in the untreated group (966.73 ± 18.42 pg/mL), significantly elevated compared with the negative control group (267.03 ± 10.96 pg/mL; *P* < 0.0001), which confirmed sustained sympathetic hyperactivity consequent to T1DM-induced cardiac autonomic dysfunction.

Compared with the untreated group, NA was significantly lower in the insulin (664.87 ± 13.44 pg/mL; *P* < 0.0001; 31.2% reduction) and the MCC950 group (875.83 ± 9.63 pg/mL; *P* < 0.0001; 9.4% reduction). However, NA concentrations in the MCC950 group remained significantly higher than those in the insulin group (*P* < 0.0001), which indicated a more modest attenuation of sympathetic hyperactivity compared with insulin therapy.

### The effects of T1DM and MCC950 on C-peptide and pancreatic β-cell function

**Figure 4:**
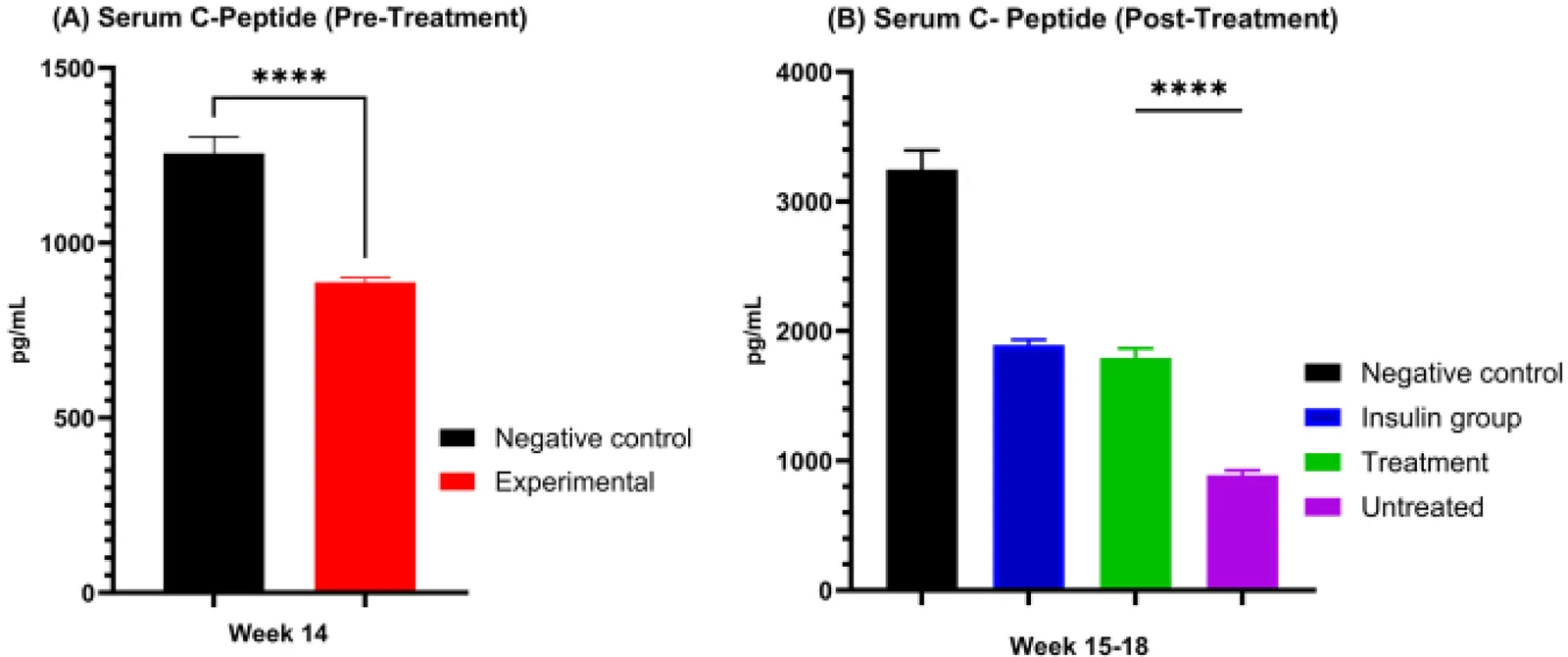
Serum C-peptide concentrations in streptozotocin-induced diabetic rats before (**A**) and after treatment (**B**). Data are presented as mean ± standard deviation (****P < 0.001). NC – negative control; PC – positive control.

Serum C-peptide concentrations were measured at two time points to evaluate endogenous insulin secretion and pancreatic β-cell function across experimental groups. C-peptide concentrations were significantly lower in the experimental group (STZ-induced, 900 pg/mL) than in the negative control group (1250 pg/mL, *P* < 0.0001), confirming successful STZ-induced β-cell destruction (Figure 4A). This 28% reduction validates T1DM induction, consistent with published reports that STZ administration at 50 mg/kg significantly impairs endogenous insulin secretion (12,21).

One-way ANOVA revealed a highly significant effect of treatment group on C-peptide levels (F[3,20] = 729.3, *P* < 0.0001). C-peptide concentration was significantly higher in the negative control group (3200 pg/mL) than in the untreated diabetic group (800 pg/mL; *P* < 0.0001; a 75% reduction). This is consistent with progressive STZ-induced β-cell destruction (Figure 4B).

In both the insulin and MCC950 groups, C-peptide was partially restored compared with the untreated group, remaining below negative control levels but significantly higher than in the untreated group (*P* < 0.0001). Homogeneity of variance was confirmed by the Brown-Forsythe test (F [3,20] = 1.553, *P* = 0.2319).

### The effects of T1DM and MCC950 on pancreatic tissue

**Figure 5:**
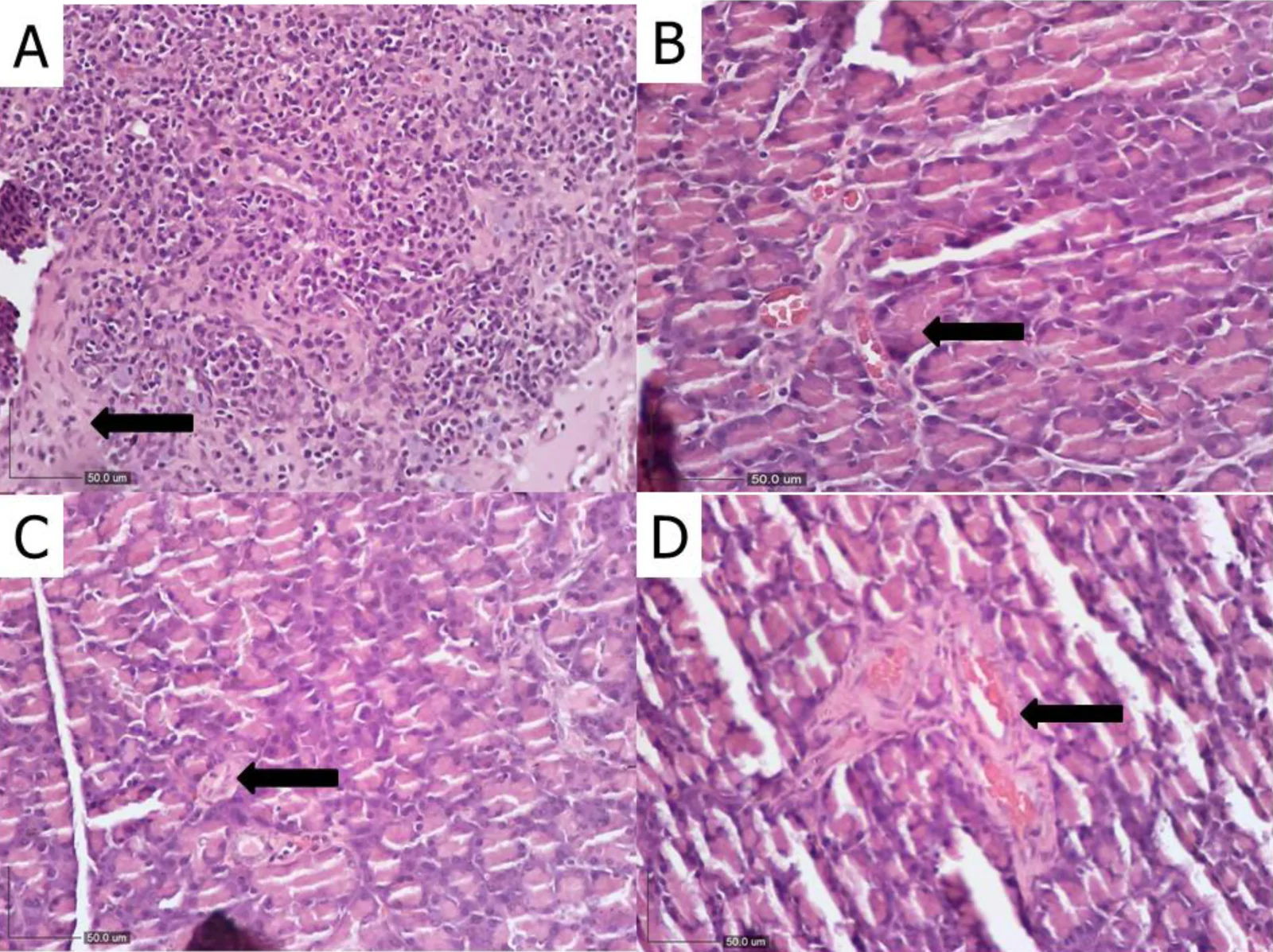
Representative hematoxylin and eosin-stained sections of the pancreas from all experimental groups (200 × magnification; scale bar = 50 μm). Black arrows indicate the islets of Langerhans. (**A**) The negative control group exhibited well-preserved pancreatic morphology with intact islets and organized acinar tissue. (**B**) The insulin-treated group showed moderately preserved pancreatic structure with minor histological alterations. (**C**) The MCC950-treated group demonstrated maintained islet morphology and orderly acinar arrangement with minimal tissue disruption. (**D**) The untreated diabetic group displayed marked pancreatic damage characterized by islet shrinkage, acinar disorganization, and prominent inflammatory cell infiltration.

The analysis of hematoxylin and eosin-stained pancreatic sections showed differences in islet morphology and exocrine tissue architecture across all four experimental groups (Figure 5). The negative control group displayed healthy pancreatic architecture characterized by well-defined islets of Langerhans, intact and uniformly arranged acinar tissue, and an absence of inflammatory infiltration or degenerative change. The insulin group exhibited mild histological alterations, including slight disruption of islet boundaries, scattered inflammatory cells, and minor acinar disorganization, though overall tissue architecture remained largely preserved. The MCC950 group demonstrated considerably better tissue preservation than the untreated diabetic group, with relatively intact islet morphology, orderly acinar arrangement, and minimal inflammatory infiltration, indicative of a protective effect of NLRP3 inflammasome inhibition on pancreatic tissue integrity. The untreated diabetic group exhibited the most severe histological damage, characterized by grossly distorted islet architecture, extensive acinar disorganization, widened interstitial spaces, heavy inflammatory cell infiltration, and focal cellular degeneration, consistent with the destructive consequences of uncontrolled hyperglycemia on pancreatic structure.

These observations were confirmed by semiquantitative scoring (Table 2). Two-way ANOVA identified significant effects of treatment group (F[3,84] = 214.6, *P* < 0.0001), histological domain (F[2,84] = 8.43, *P* < 0.001), and their interaction (F [6,84] = 3.17, *P* = 0.0073). The untreated group had the highest scores across all domains: islet morphology, acinar integrity, inflammatory infiltration, and composite injury, each significantly exceeding those of the negative control, insulin, and MCC950 groups (*P* < 0.001). MCC950 treatment significantly reduced injury scores across all domains compared with no treatment (*P* < 0.01) and produced a lower composite score (2.38 ± 0.52) than that reported in the insulin group (3.75 ± 0.89; *P* < 0.05), which suggests that direct NLRP3 inhibition confers greater pancreatic tissue protection than glycemic correction alone. The negative control group exhibited the lowest scores throughout, consistent with normal pancreatic morphology.

**Table 2:** Semiquantitative histological scores of pancreatic tissue across experimental groups*

Groups	Islet morphology	Acinar tissue integrity score	Inflammatory infiltration score	Total composite score
**Negative control**	0.25 ± 0.16	0.13 ± 0.10	0.25 ± 0.16	0.63 ± 0.28
**Insulin**	1.38 ± 0.35	1.13 ± 0.35	1.25 ± 0.46	3.75 ± 0.89
MCC950 t**reatment**	0.88 ± 0.23^†^	0.75 ± 0.27^†^	0.75 ± 0.27^†^	2.38 ± 0.52^†^
**Untreated**	2.88 ± 0.44	2.75 ± 0.46	2.88 ± 0.35	8.50 ± 0.93

*Values are expressed as mean ± standard deviation (n = 8-10 per group).

†*P* < 0.0001 compared with the untreated group.

### The effects of T1DM and MCC950 on fasting serum lipids

**Figure 6:**
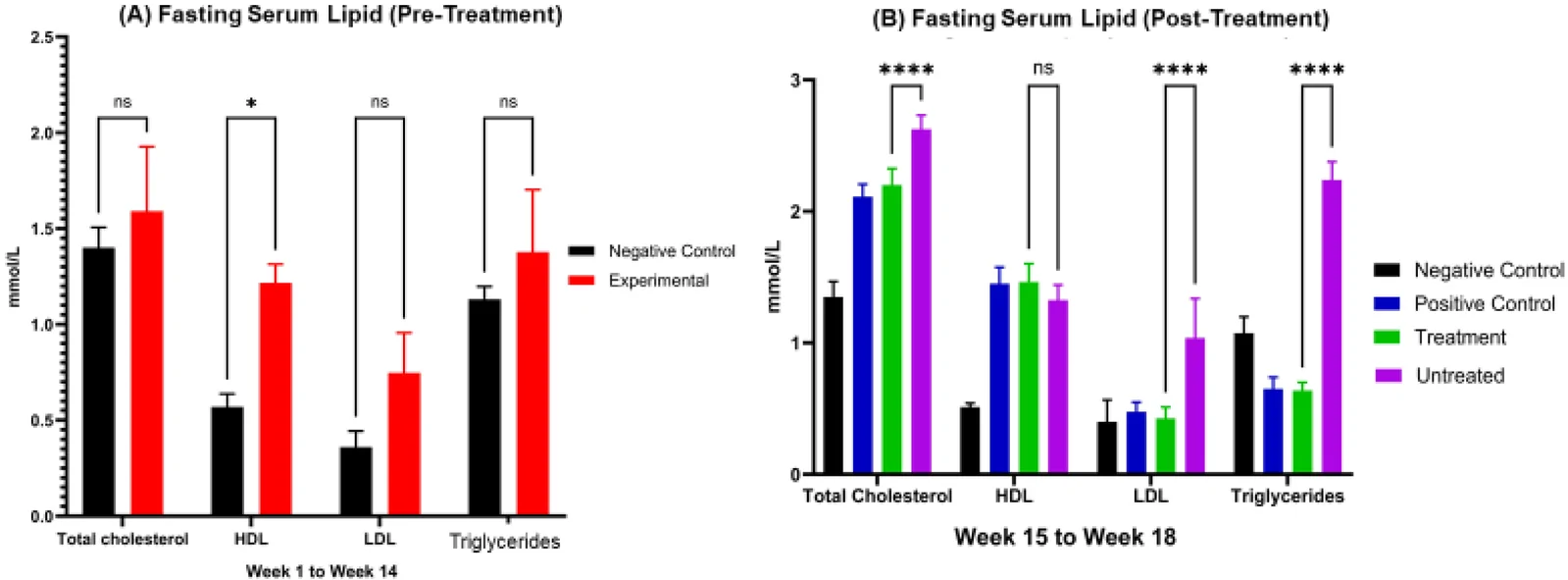
Fasting serum lipid before (**A**) and after treatment (**B**). All the lipid parameters non-significantly (ns) increased in the experimental group during the pre-treatment period, except for an increase in HDL levels (*P < 0.05). Following treatment, MCC950 significantly reduced total cholesterol (****P < 0.0001), low-density lipoprotein (LDL) (****P < 0.0001) and triglycerides (TG) (****P < 0.0001) in type I diabetic cardiovascular autonomic neuropathy rats but did not significantly affect high-density lipoprotein (HDL). Additionally, MCC950 significantly improved TG levels (****P < 0.0001) compared with insulin treatment.

In the pre-treatment period, TC level was highest compared with HDL, LDL, and TG levels. Only HDL significantly increased in the experimental group (Figure 6, left panel). At week 14, all four lipid parameters decreased in both the insulin and MCC950 groups. As anticipated, all lipid parameters persistently increased in the untreated group. Surprisingly, only LDL and TG were significantly reduced in the MCC950 group compared with the untreated group, while only TG were significantly reduced in the MCC950 group compared with the insulin group (Figure 6, right panel).

## DISCUSSION

In this study, MCC950 treatment produced beneficial metabolic and biochemical effects in STZ-induced T1DM with sympathetic dysfunction. Specifically, MCC950 significantly reduced fasting blood glucose, LDL, and TG levels compared with untreated diabetic rats, while TC and HDL levels remained unchanged. It also partially restored serum C-peptide levels and preserved pancreatic tissue architecture, accompanied by a reduction in serum NA, indicating attenuation of sympathetic dysregulation. Notably, the reduction in LDL and TG, together with the improvement in pancreatic histological features, suggests that NLRP3 inflammasome inhibition may exert beneficial effects beyond glycemic control alone. However, because direct functional assessments of cardiac autonomic function and molecular confirmation of NLRP3 inflammasome inhibition were not performed, these findings should be interpreted as evidence of improved metabolic parameters and attenuation of sympathetic dysfunction rather than of definitive reversal of DCAN.

Successful induction of T1DM was confirmed by a marked elevation in fasting blood glucose following STZ administration. These findings are consistent with the well-established diabetogenic action of STZ, which selectively destroys pancreatic β-cells through DNA alkylation, oxidative stress generation, and mitochondrial dysfunction (21,22).

Although insulin significantly reduced fasting blood glucose compared with no treatment, glucose concentrations remained above those of the non-diabetic controls. This observation is consistent with previous experimental studies using moderate-dose insulin replacement, where partial rather than complete glycemic correction was often achieved to avoid hypoglycemia and excessive mortality in STZ-induced diabetic animals (23,24). Consequently, the insulin-treated group in the present study should be regarded as representing partial rather than complete restoration of normoglycemia, and the downstream effects should be interpreted within this context (25).

An important finding of the present study was significantly elevated NE concentrations in diabetic animals, indicating substantial sympathetic dysregulation. Elevated circulating NE levels have been reported in both experimental and clinical studies of diabetic autonomic neuropathy and are believed to reflect compensatory sympathetic activation during the early stages of autonomic injury (26,27). Prolonged sympathetic dysregulation may contribute to oxidative stress, impaired baroreflex sensitivity, myocardial injury, and progression of DCAN.

Assessment of serum C-peptide further confirmed substantial pancreatic β-cell dysfunction. Untreated diabetic animals exhibited markedly lower C-peptide concentrations than the negative control group. This finding is consistent with extensive STZ-induced β-cell destruction and severe impairment of endogenous insulin production (28). In contrast, the insulin and MCC950 groups demonstrated partially restored C-peptide concentrations. Importantly, as exogenous insulin does not contain C-peptide, it cannot directly increase circulating C-peptide concentrations (5). Consequently, higher C-peptide levels observed in the insulin group most likely reflected a preserved or improved function of residual surviving pancreatic β-cells following improved glycemic control (29), with reduced glucotoxicity, oxidative stress, and inflammatory stress allowing these remaining β-cells to recover part of their secretory capacity (30). However, because STZ destroys β-cells, C-peptide concentrations remained markedly lower than those in non-diabetic controls, which indicates only partial preservation of endogenous insulin secretion rather than β-cell regeneration (31). This also suggests that both interventions preserved residual β-cell function and improved endogenous insulin secretory capacity. These findings support previous reports demonstrating that maintenance of β-cell integrity may attenuate metabolic dysfunction and delay the progression of diabetic complications (32).

The present histological findings demonstrated marked pancreatic structural deterioration in untreated diabetic rats, characterized by disruption of islet architecture, acinar disorganization, inflammatory infiltration, and increased composite injury scores. These observations are consistent with the known pathological consequences of persistent hyperglycemia: promoted oxidative stress, inflammatory activation, and progressive pancreatic tissue injury (33). In contrast, treatment with MCC950 substantially preserved pancreatic morphology, maintaining islet integrity, improving acinar organization, and reducing inflammatory cell infiltration. Notably, semiquantitative analysis showed significantly lower histological injury scores in the MCC950 group compared with untreated diabetic animals and, in several domains, lower scores than those observed in the insulin-treated group. These findings suggest that inhibition of the NLRP3 inflammasome may be associated with attenuation of pancreatic inflammatory changes beyond metabolic correction alone. However, because the current study did not perform direct molecular assessment of inflammasome activity and inflammatory mediators, the mechanisms underlying these histological improvements remain speculative. Therefore, while the present findings support a potential protective association between MCC950 treatment and pancreatic tissue preservation in experimental diabetes, further studies incorporating molecular and functional validation are required to confirm whether these effects are directly mediated through NLRP3 signaling pathways. The present study assessed multiple complementary parameters to support diabetic model establishment, while autonomic involvement was evaluated independently using biochemical evidence associated with autonomic alteration rather than blood glucose alone.

This study indicates the potential of MCC950 as a dual-targeted therapeutic agent for managing dyslipidemia and DCAN in T1DM. Through comprehensive evaluation of lipid profiles and autonomic function, MCC950 significantly reduced LDL and TG levels in diabetic rats, addressing key components of dyslipidemia associated with diabetes. These findings underscore the importance of targeting the NLRP3 inflammasome pathway in mitigating dyslipidemia, thereby offering new avenues for therapeutic intervention in diabetes-related complications (34).

The selective impact of MCC950 on TG and LDL levels while leaving other lipid parameters unchanged could stem from its specific mechanism of action. This selectivity suggests that MCC950 might exert its effects through modulating inflammatory pathways that are particularly relevant to TG and LDL regulation, while sparing or minimally affecting pathways governing HDL and TC levels (35-37). Understanding the differential impact of MCC950 highlights potential avenues for targeted therapeutic interventions in conditions characterized by dyslipidemia and inflammation.

Furthermore, our study provides insights into the underlying mechanisms by which MCC950 affects dyslipidemia and DCAN (38). By elucidating the molecular pathways involved in inflammation and metabolism, we contributed to a better understanding of the pathophysiology of these conditions and the potential therapeutic targets for intervention. The observed improvements in glucose and lipid parameters following MCC950 treatment offer valuable evidence supporting its efficacy as a dual-targeted therapy, highlighting its potential clinical utility in managing diabetic complications.

The efficacy of short-duration MCC950 treatment in reducing lipid and sugar parameters in Wistar rats with type 1 DCAN could be attributed to its potent anti-inflammatory properties and their rapid impact on metabolic pathways. By mitigating inflammation, MCC950 may alleviate insulin resistance, enhance glucose uptake, and improve lipid metabolism within a relatively short timeframe (10). Additionally, the rapid response to MCC950 treatment may also reflect its ability to modulate key signaling pathways involved in metabolic homeostasis, such as AMP-activated protein kinase (39) and peroxisome proliferator-activated receptor gamma (40), which further contributed to the observed reductions in lipid and sugar parameters. The findings emphasize a significant role of inflammation in exacerbating metabolic dysregulation in DCAN, highlighting the therapeutic potential of targeting the NLRP3 inflammasome to mitigate diabetic complications. Future investigations should incorporate heart rate variability analysis, autonomic functional testing, and histological assessment to strengthen confirmation of autonomic dysfunction.

This study is limited by the reliance on animal models, which may not fully recapitulate the complexity of human DCAN. Additionally, MCC950, targeting the NLRP3 inflammasome pathway, may not encompass the entirety of inflammatory pathways implicated in DCAN. Although insulin-treated animals had significantly reduced fasting blood glucose compared with untreated diabetic animals, glycemia was not completely normalized. Persistent hyperglycemia may have therefore continued to contribute to metabolic disturbances and biomarkers of sympathetic dysfunction. Future studies employing optimized insulin regimens or intensive glycemic control protocols are warranted. Although elevated serum NA is an established biomarker of sympathetic dysfunction and has been used to support DCAN development in experimental diabetes models, cardiac autonomic neuropathy (eg, heart rate variability, baroreflex sensitivity, electrocardiography, or cardiac autonomic nerve histology) was not directly validated. Therefore, the present findings should be interpreted as evidence of improved metabolic parameters and attenuation of sympathetic dysfunction rather than definitive reversal of DCAN. Moreover, the absence of long-term follow-up limits the assessment of MCC950’s sustained efficacy and safety profile.

In conclusion, our findings suggest that NLRP3 inflammasome inhibition may have therapeutic potential in improving metabolic abnormalities and sympathetic dysregulation associated with experimental T1DM. However, as direct functional, histological, and immunohistochemical assessments of cardiac autonomic neuropathy were not performed, no definitive conclusions can be drawn regarding the effects of MCC950 on diabetic cardiac autonomic neuropathy. Further mechanistic and functional studies are warranted to confirm these findings and clarify the role of MCC950 in diabetes-associated autonomic dysfunction.
